# Elicitor Activity of Low-Molecular-Weight Alginates Obtained by Oxidative Degradation of Alginates Extracted from *Sargassum muticum* and *Cystoseira myriophylloides*

**DOI:** 10.3390/md21050301

**Published:** 2023-05-16

**Authors:** Meriem Aitouguinane, Zainab El Alaoui-Talibi, Halima Rchid, Imen Fendri, Slim Abdelkafi, Mohamed Didi Ould El-Hadj, Zakaria Boual, Didier Le Cerf, Christophe Rihouey, Christine Gardarin, Pascal Dubessay, Philippe Michaud, Guillaume Pierre, Cédric Delattre, Cherkaoui El Modafar

**Affiliations:** 1 Centre d’Agrobiotechnologie et Bioingénierie, Unité de Recherche Labellisée CNRST (Centre AgroBiotech, URL-CNRST 05), Faculté des Sciences et Techniques, Université Cadi Ayyad, Marrakech 40000, Morocco; meriem.aitouguinane@etu.uca.fr (M.A.); elmodafar@uca.ac.ma (C.E.M.); 2 Clermont Auvergne INP, CNRS, Institut Pascal, Université Clermont Auvergne, F-63000 Clermont-Ferrand, France; christine.gardarin@uca.fr (C.G.); pascal.dubessay@uca.fr (P.D.); philippe.michaud@uca.fr (P.M.); 3 Laboratoire de Biotechnologies et Valorisation des Ressources Végétales, Faculté des Sciences, Université Chouaib Doukkali, El Jadida 24000, Morocco; rchidhalima@hotmail.com; 4 Laboratoire de Biotechnologie des Plantes Appliquée à l’Amélioration des Cultures, Faculté des Sciences de Sfax, Université de Sfax, Sfax 3000, Tunisia; imen.fendri@fss.usf.tn; 5 Laboratoire de Génie Enzymatique et de Microbiologie, Equipe de Biotechnologie des Algues, Ecole Nationale d’Ingénieurs de Sfax, Université de Sfax, Sfax 3000, Tunisia; slim.abdelkafi@enis.tn; 6 Laboratoire de Protection des Ecosystèmes en Zones Arides et Semi-Arides, Faculté des Sciences de la Nature et de la vie BP 511, Université Kasdi Merbah de Ouargla, Ouargla 30000, Algeria; mohameddidi@yahoo.fr (M.D.O.E.-H.); biozakaria1983@gmail.com (Z.B.); 7 Polymères Biopolymères Surfaces, Normandie Université, UNIROUEN, INSA Rouen, CNRS, UMR6270, F-76821 Mont Saint-Aignan, France; didier.lecerf@univ-rouen.fr (D.L.C.); christophe.rihouey@univ-rouen.fr (C.R.); 8 Institut Universitaire de France (IUF), 1 Rue Descartes, F-75005 Paris, France

**Keywords:** alginates, low-M_w_ alginates, elicitor, seaweeds, tomato seedlings

## Abstract

Alginates extracted from two Moroccan brown seaweeds and their derivatives were investigated for their ability to induce phenolic metabolism in the roots and leaves of tomato seedlings. Sodium alginates (ALSM and ALCM) were extracted from the brown seaweeds *Sargassum muticum* and *Cystoseira myriophylloides*, respectively. Low-molecular-weight alginates (OASM and OACM) were obtained after radical hydrolysis of the native alginates. Elicitation was carried out by foliar spraying 20 mL of aqueous solutions (1 g/L) on 45-day-old tomato seedlings. Elicitor capacities were evaluated by monitoring phenylalanine ammonia-lyase (PAL) activity, polyphenols, and lignin production in the roots and leaves after 0, 12, 24, 48, and 72 h of treatment. The molecular weights (M_w_) of the different fractions were 202 kDa for ALSM, 76 kDa for ALCM, 19 kDa for OACM, and 3 kDa for OASM. FTIR analysis revealed that the structures of OACM and OASM did not change after oxidative degradation of the native alginates. These molecules showed their differential capacity to induce natural defenses in tomato seedlings by increasing PAL activity and through the accumulation of polyphenol and lignin content in the leaves and roots. The oxidative alginates (OASM and OACM) exhibited an effective induction of the key enzyme of phenolic metabolism (PAL) compared to the alginate polymers (ALSM and ALCM). These results suggest that low-molecular-weight alginates may be good candidates for stimulating the natural defenses of plants.

## 1. Introduction

Alginate is an anionic polymer present in the cell walls of brown seaweeds, representing more than 40% of their dry weight [1]. It is one of the most abundant biopolymers [2]. An estimated 23,000 tons of alginates are produced annually from 85,000 tons of brown algae [1]. Furthermore, the market for these polysaccharides is expected to grow significantly by 2025, reaching USD 923.8 million [3]. Alginate is mainly composed of two conformational isomer residues, d-mannuronic acid (M) and l-guluronic acid (G), constituting homopolymeric (MM, GG) and heteropolymeric (MG, GM) sequential block structures [4]. Brown seaweeds, such as *Ascophyllum*, *Durvillaea*, *Ecklonia*, *Laminaria*, *Lessonia*, *Macrocystis*, and *Sargassum*, are recognized as the main source of commercial alginate [3]. Due to its physicochemical properties, environmental biocompatibility, biodegradability, and nontoxicity, alginate is considered an ideal biopolymer that could be used in a variety of applications. This polysaccharide has been widely used in the food [5], pharmaceutical, biomedical [6], and industrial fields [7]. In addition, several studies have reported the use of alginate in agriculture to stimulate plant growth and development [8,9] and to induce plant resistance mechanisms [10,11,12].

The application of alginates as elicitors can sometimes be limited by their low solubility in water and viscosity when high concentrations are required. For this reason, producing low-molecular-weight (LMW) alginates or oligoalginates is a good way to obtain new functional derivatives with better solubility and bioavailability. This enables the preparation of high concentrations without increasing viscosity. LMW alginates can be obtained using several methods, including physical, chemical, and enzymatic degradation [13]. Today, chemical methods, such as acid, alkaline, and oxidative hydrolysis, are the simplest, most effective, least expensive, and most reproducible on an industrial scale. Compared to acid degradation, radical depolymerization using H_2_O_2_ as an oxidizing agent is a green and eco-friendly processor that produces high-recovery LMW alginates without generating toxic residues, as the only by-molecule is H_2_O.

Recently, low-molecular-weight alginates (LMW), including oligomers, have gained much attention among researchers for their immunomodulatory [14], antimicrobial [15,16], antitumor [17], antioxidant [18,19], prebiotic [20], and antidiabetic activities [21]. In agriculture, low-M_w_ alginates have been recognized as fertilizers, soil conditioners, and inducers of the natural defenses of plants [9,11,22,23,24]. The use of elicitors in crop protection is a new approach that could contribute to greater agricultural sustainability by minimizing chemical product applications and optimizing biocontrol strategies. Several studies have investigated some functional mechanisms of oligoalginates in plants [25,26]. Recently, tomato seedlings and date palm roots treated with oligoalginates from *Bifurcaria bifurcata* showed the induction of natural defenses by increasing PAL activity and the production of polyphenols [11,12]. This has been accompanied by the induction of gene expression and resistance to *Fusarium oxysporum* f. sp. *albedinis* [12]. Further works have revealed the ability of alginate oligomers to overcome abiotic stress [23,26,27].

Oligosaccharides involved in plant–pathogen interactions are originally produced through degradation of the cell wall polysaccharides of microorganisms or plants. The generated oligomers are then recognized as PAMPs (pathogen-associated molecular patterns), MAMPs (microbe-associated molecular patterns), or DAMPs (damage-associated molecular patterns) [28,29]. After signal transduction, a cascade of defense responses is activated. Systemic acquired resistance (SAR) is a distinct signal transduction pathway that plays an important role in plant resistance by activating enzymes responsible for secondary metabolite synthesis, leading to resistance to a wide range of phytopathogens [30].

In the present study, alginates isolated from *Sargassum muticum* (ALSM) and *Cystoseira myriophylloides* (ALCM) and low-M_w_ alginate derivatives (OACM and OASM) were investigated for their ability to induce phenolic metabolism in the leaves and roots of tomato seedlings. OASM and OACM were obtained after the radical hydrolysis of the alginate polymers. 

## 2. Results and Discussion

### 2.1. Structural Analysis of Alginate Extracted from S. muticum and C. myriophylloides and Their Derivatives

**Figure 1:**
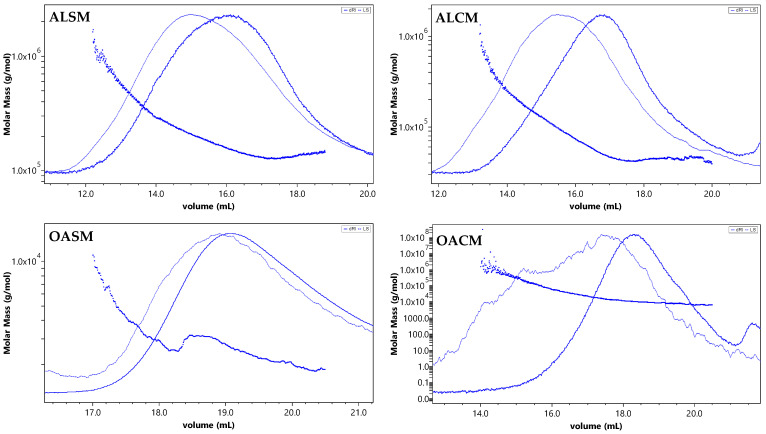
SEC-MALLS chromatograms and molecular weight distributions of alginates and low-M_w_ alginates in 0.1 M LiNO_3_. Light scattering signal (LS) (dashed line); differential refractive index signal (dRI) (full line); molecular weight distribution (bold line); injection volume (100 µL).

Two alginates, ALSM and ALCM, were extracted and purified from the Moroccan brown seaweeds *S. muticum* and *C. myriophylloides*, respectively. To prepare the low-molecular-weight alginates OACM and OASM, ALCM and ALSM, respectively, were oxidized using H_2_O_2_. As described previously [10], the average molecular weights (M_w_) of ALCM and ALSM are 76 kDa and 202 kDa, respectively. The OACM derivative was characterized by a M_w_ of 19 kDa, while the OASM showed a low M_w_ of 3 kDa. Figure 1 shows the SEC-MALLS chromatograms of each fraction. Both alginate polymers (ALSM and ALCM) exhibited monomodal symmetrical curves with intense light scattering signals at low elution volumes. This indicates that the fractions contained molecules of similar molecular weights. However, the SEC curves changed from unsymmetrical after oxidative degradation, especially for OACM, presenting a strong light scattering signal at high elution volumes (Figure 1).

**Figure 2:**
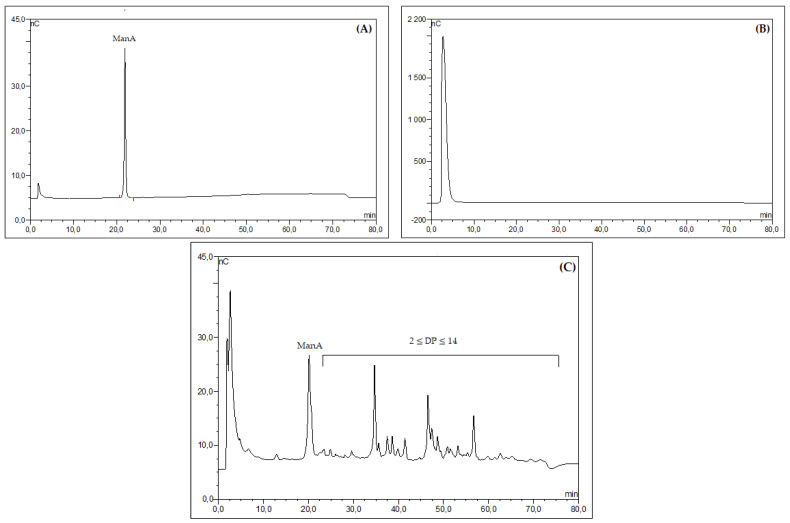
HPAEC-PAD analysis of OACM (**B**) and OASM (**C**) derivatives obtained after 6 h of radical hydrolysis of alginates (ALSM and ALCM) using H_2_O_2_; (**A**) HPAEC-PAD profile of mannuronic acid (standard monosaccharide).

HPAEC-PAD analysis of both alginates confirmed that ALSM was depolymerized to the oligoalginate OASM with a 2 ≤ DP (degree of polymerization) ≤ 14 (Figure 2C), while ALCM was partially hydrolyzed, generating the low-molecular-weight alginate OACM with a DP = 88 (Figure 2B). The DPs were calculated based on the M_w_ of the generated fractions and the M_w_ of repeated units of guluronic acid or mannuronic acid (216 g·mol^−1^). 

To obtain low-molecular-weight alginates, various methods have been used, such as enzymatic depolymerization [10], acid hydrolysis, and the oxidation method [31]. During the enzymatic degradation of alginate by alginate lyases, unsaturated, nonreducing terminal residues are formed, which are not always desirable [19]. Furthermore, the main issues with this process are its high cost and long reaction time requirements. Acid hydrolysis using sulfuric acid (H_2_SO_4_) [32] and hydrochloric acid (HCl) [31] is a simple and easy method, but it involves toxic residues in the final products that are difficult to remove. Moreover, it generates a low yield of derivatives [31]. Hydrogen peroxide oxidation has become an eco-friendly alternative for alginate degradation, with a recovery rate of 87% [31]. In this study, the yields of the OASM and OACM fractions were approximately 82% and 81.26%, respectively. The rate and the M_w_ of the derived fractions depended on alginate structure, reaction time, temperature, pH, and hydrogen peroxide (H_2_O_2_) concentration [33]. Hydrogen peroxide hydrolysis consists of the detachment of hydrogen atoms at all ring C-H bonds of polysaccharides. Hydroxyl radical attack leads to the formation of carboxyl groups in two ways. The first is the oxidation of -OH at C6 to carbonyl groups and then to COOH, and the second is the breakdown of glycosidic bonds by the action of generated radicals at the C1 position, forming a terminal carboxyl [34,35,36]. Several studies have used H_2_O_2_ to oxidize polysaccharides, including alginate [31,34,37], amylose [38], κ-carrageenan [39], chitosan [40], gellan gum [36], and cellulose [41]. In this study, despite its high M_w_ (202 kDa), ALSM was hydrolyzed faster than ALCM (76 kDa) under the same conditions. This could be explained by resistance to the hydroxyl radical attack due to the presence of sulfates (7.29%) in ALCM (fucoidan contamination) [10]. Moseley et al. reported that sulfated glycosaminoglycans (GAG) were less susceptible to degradation and chemical modification compared to non-sulfated GAG, suggesting that the presence of sulfate could protect molecules against reactive oxygen species (ROS) hydrolysis [42]. It has been reported that sulfate content has an effect on scavenging-generated hydroxyl radicals [43]. Further studies are needed to develop and confirm this hypothesis. 

**Figure 3:**
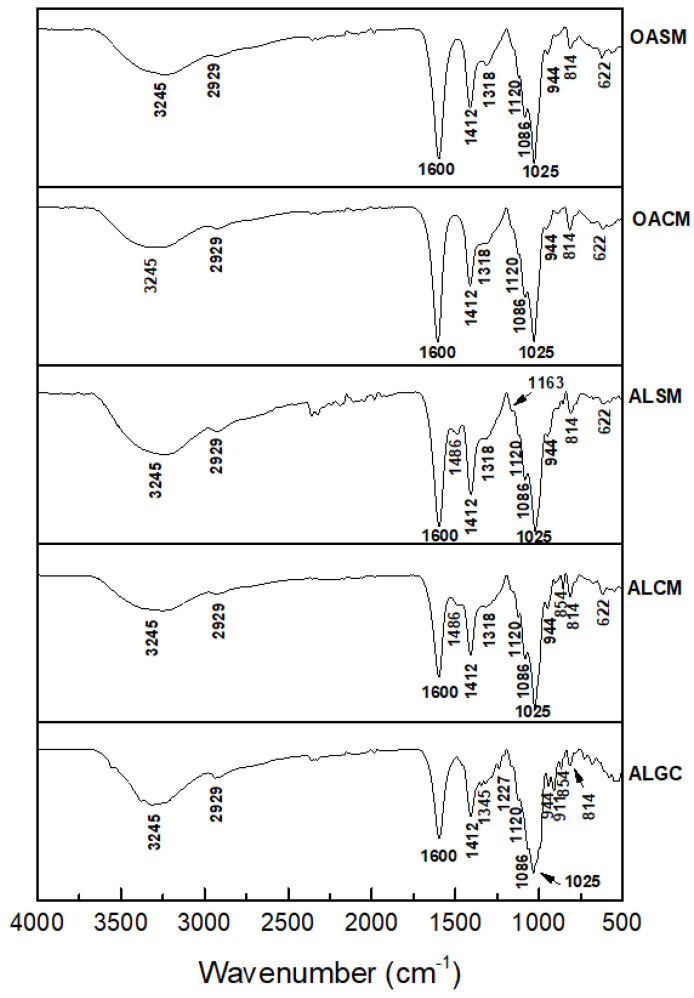
FTIR spectra of native alginates extracted from *C. myriophylloides* (ALCM) and *S. muticum* (ALSM) commercial alginates (ALGC) and oxidized alginates (OACM and OASM).

In order to analyze the changes in the composition and structure of oxidized alginate, FTIR analysis was performed. The spectra were compared to unmodified alginates, including commercial alginate (ALGC), as shown in Figure 3. The native alginates and their derivatives presented characteristic bands at approximately 1600, 1412, 1086, and 1025 cm^−1^. Peaks at 3245 and 2929 cm^−1^ were attributed to -OH and -CH stretching bonds, respectively. The two intense bands at 1600 and 1412 cm^−1^ corresponded to asymmetric and symmetric stretching vibrations of carboxyl groups (COOH) of alginate, respectively [44]. Taking the peak at 1600 cm^−1^ as the reference, the COOH groups did not change after the oxidation of native alginates (ALCM and ALSM) (Figure 3). The weak bands at 1086 cm^−1^ and strong peaks at 1025 cm^−1^ may be assigned to the C=O and C-C bonds of guluronic and mannuronic units, respectively [44,45]. Signals at 944 cm^−1^ were assigned to the C=O stretching vibration of uronic acids [45]. The characteristic bands of *β*-mannuronic acid residues are located at 814 cm^−1^ [44]. When compared to ALGC, the positions of the characteristic bands of ALCM and ALSM are similar. No obvious differences were reported in the FTIR spectra of the alginates and their derivatives (OACM and OASM). These results indicate that the depolymerization of H_2_O_2_ with a ratio of 1:1 (*w*/*w*) under the conditions described above allows for the breakdown of glycosidic bonds in alginate, keeping its main chain and structure unchanged. It has been reported that H_2_O_2_ treatment under extreme conditions changes the structure, with the appearance of a new band at 1730 cm^−1^, indicating the introduction of carboxyl and carbonyl groups to the structure of polysaccharides. The peak became more intense with the increase in oxidation degree [34,36,39]. 

### 2.2. Effect of Alginates and Their Derivatives on Phenolic Metabolism in Tomato Seedlings

Alginates extracted from the brown seaweeds *C. myriophylloides* (ALCM) and *S. muticum* (ALSM) and their derivatives were applied by foliar spray on 45-day-old tomato seedlings to evaluate their elicitor effects. The systemic responses were investigated by measuring phenylalanine ammonia-lyase (PAL) activity, phenolic compounds, and lignin content in the roots and upper leaves (n and n − 1) after 0, 12, 24, 48, and 72 h. The control plants were treated with distilled water.

#### 2.2.1. PAL Activity

PAL activity was significantly induced in tomato roots and leaves after treatment with alginates and their derivatives, depending on incubation time (*p* < 0.05).

**Figure 4:**
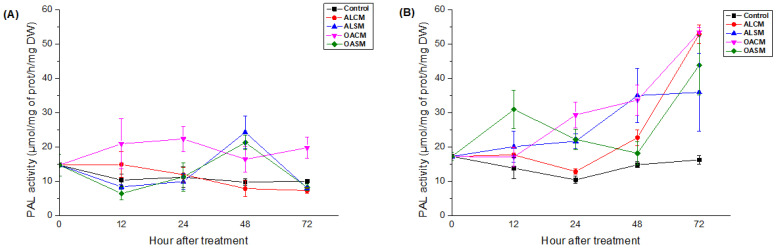
Effect of alginates (ALCM and ALSM) and their derivatives (OACM and OASM) on PAL activity in the upper leaves (**A**) and roots (**B**) of tomato seedlings. Each value is the mean of four repetitions ± standard error (SE).

In comparison to the native polymers (ALCM and ALSM), foliar spray application of the hydrolyzed alginates (OACM and OASM) showed an earlier onset of PAL activity. Plants stimulated with OACM manifested an increase in PAL levels as early as 12 h in the leaves and 24 h in the roots, which remained high throughout the incubation period (Figure 4). In response to OASM, levels rose steadily from 12 h in the roots, reaching a peak value after 72 h of elicitation. Additionally, the decrease in PAL levels in roots was accompanied by their increase in leaves after 48 h (Figure 4). The responses were expressed after 24 h in the roots of seedlings treated with ALSM and 48 h after the application of ALCM (Figure 4B). Levels increased in the leaves after 48 h of ALSM elicitation. PAL activity in the leaves was not affected by ALCM treatment (Figure 4A).

#### 2.2.2. Phenolic Compounds

**Figure 5:**
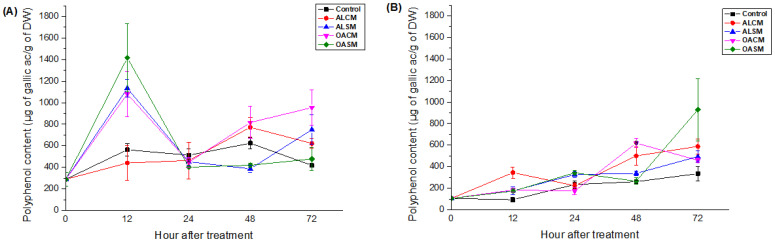
Effect of alginates (ALCM and ALSM) and their derivatives (OACM and OASM) on the accumulation of polyphenols in the upper leaves (**A**) and roots (**B**) of tomato seedlings. Each value is the mean of four repetitions ± standard error (SE).

The effect of alginates and their derivatives on the accumulation of phenolic compounds is presented in Figure 5. The responses were dependent on the duration after elicitation (*p* < 0.05).

The foliar spray of ALSM, OACM, and OASM significantly induced polyphenol production in the leaves after 12 h (Figure 5A). Levels declined sharply in the following days, except for plants treated with OACM, which showed another upward trend after 72 h (Figure 5A). In root tissues, ALSM and its oligomers followed similar patterns. Initially, levels rose after 24 h and then declined sharply after 48 h, reaching control values. After 72 h, polyphenol content significantly increased to rise toward its peak (Figure 5B). The effects of ALCM on the synthesis of these compounds in the leaves were not observed. However, they manifested elicitor activity after 12, 48, and 72 h in the roots. Phenolic content was promoted in the roots of the tomato plants after 48 and 72 h of elicitation by OACM (Figure 5B).

#### 2.2.3. Lignin Content

**Figure 6:**
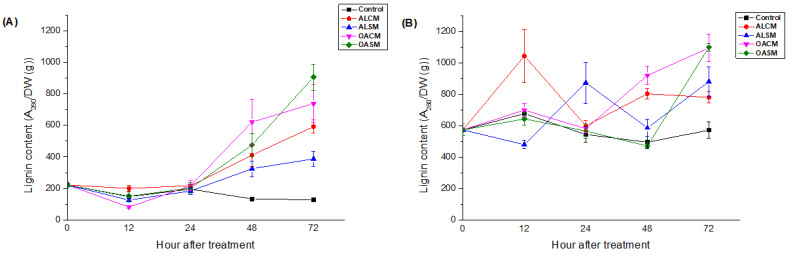
Effect of alginates (ALCM and ALSM) and their derivatives (OACM and OASM) on lignin content in the upper leaves (**A**) and roots (**B**) of tomato seedlings. Each value is the mean of four repetitions ± standard error (SE).

As illustrated in Figure 6, all fractions showed their ability to enhance the synthesis of lignin content in the root and leaf tissues of tomato seedlings, depending on the duration time (*p* < 0.05). Lignin content increased in the leaves 48 h after foliar treatment. This was accompanied by a decrease in polyphenol content (Figure 5A), meaning that the phenolic compounds were probably used as precursors to lignin biosynthesis. It is known that lignin is formed via the oxidative polymerization of monolignols (sinapyl alcohol, coniferyl alcohol, and *p*-coumaryl) coming from the phenylpropanoid pathway [46]. Other phenolic monomers, such as hydroxycinnamaldehydes, tricin, and hydroxystilbenes, have also been reported to form lignin subunits [47,48,49].

When compared to their native polymers, the alginate derivatives OACM and OASM exhibited important production of lignin content in the roots and leaves after 72 h (Figure 6). After 12, 24, 48, and 72 h, respectively, the first elicitor activity of ALCM, ALSM, OACM, and OASM was detected in the roots (Figure 6B). When compared to their derivatives, the native alginates showed earlier production of lignin (Figure 6B).

In this study, alginates and their oxidized derivatives triggered PAL activity and the synthesis of phenolic compounds and lignin in the roots and leaves of tomato seedlings. These responses depended on the incubation time and were systemic. 

PAL is one of the most studied enzymes in plant defense responses. It initiates primary (shikimate pathway) metabolism and secondary (phenolic pathway) metabolism by catalyzing the conversion of l-phenylalanine to *trans*-cinnamic acid. This leads to the formation of the majority of phenylpropanoid compounds, including flavonoids, stilbenes, tannins, and complex polymers (lignin and suberin), via a series of methylation, dehydration, polymerization, and hydroxylation reactions [50,51]. These metabolites are then degraded by several oxidative enzymes, such as polyphenol oxidases (PPO), peroxidases (POD), laccases (LAC), and lipoxygenases (LOX) [51]. 

A significant increase in PAL, polyphenols, and lignin was observed in the tomato seedlings after treatment with lactic acid bacterium (LAB) isolated from rhizosphere soil, *Fusarium* mycelium extract, chitosan, *Fusarium* culture filtrate, *Trichoderma* mycelium extract, and salicylic acid (SA) [52,53,54]. Polysaccharides from seaweeds have also been reported as inducers of the natural defenses of apple fruits, date palms, tomato seedlings, olive leaves, and twigs [55,56,57,58,59]. Alginates derived from *S. muticum* and *C. myriophylloides*, as well as their derivatives, were found to be effective in inducing phenolic metabolism in tomato seedlings and olive leaves [10], indicating their broad-spectrum activity. A previous study reported the capacity of alginate isolated from the brown seaweeds *Bifurcaria bifurcata* and *Fucus spiralis* to induce PAL and polyphenol production in the leaves of tomato plants and the roots of date palms [11,22].

Low-molecular-weight alginates have been widely used as antimicrobial, antioxidant, immunomodulatory, prebiotic, antihypertensive, antidiabetic, anticoagulant, and antitumor agents [60]. However, their applications in agriculture as plant defense stimulators remain limited. In the present work, the oligomer OASM (3 kDa) and the low-molecular-weight alginate OACM were found to be more effective elicitors in inducing PAL activity compared to the native polymers. Similar conclusions were reached after the treatment of tomato plants with oligoalginates with a M_w_ of 5 kDa [11]. OASM fractions have also shown their effectiveness in enhancing PAL and TAL (tyrosine ammonia-lyase) activities in olive leaves compared to alginate polymers [10]. Previous studies have reported the ability of oligoalginates to stimulate defenses against biotic and abiotic stresses, as well as growth in plants [8]. Oligoalginates produced by enzymatic degradation reduced the effects of salt stress in *Brassica campestris* L. [23]. Wheat seedlings treated with exogenous alginate oligosaccharides (AOS) showed improved resistance to drought stress during the growth period by enhancing biomass, chlorophyll and proline content, and antioxidant enzyme activities [26]. The application of algino-oligosaccharides obtained using alginate lyase from *Flavobacterium* sp. has shown the induction of PAL activity and an accumulation of phytoalexin in soybean cotyledon [61]. Furthermore, these oligosaccharides have shown their protective effects against bacteria, fungi, and viruses by activating the salicylic acid pathway and the synthesis of secondary metabolites in various plants [8,62,63]. In addition, many other oligosaccharides have been reported to induce plant defenses. For example, chitosan and carrageenan-derived oligosaccharides have revealed their effects in stimulating plant defenses against a wide range of pathogens, including *Fusarium solani*, *Botrytis cinerea,* tobacco necrosis virus (TNV), *Alternaria kikuchiana*, *Physalospora piricola, Sclerotinia sclerotiorum, Lophodermium* sp., *Pseudomonas syringae* DC3000, tobacco mosaic virus (TMV), and *Peptobacterium carotovorum* [8].

Oligo-galacturonides (OGAs) and oligo-xyloglucans (OXGLs) derived from plant cell walls have been shown to be able to elicit defense responses, mainly the accumulation of ROS, activation of mitogen-activated protein kinases (MAPKs), and pathogenesis-related proteins (PR), as well as callose deposition, leading to the protection of plants against pathogen infections [64,65]. Elicitor activities are dependent on several factors, including the degree of polymerization (DP), conformation, branching characteristics, features of glycosidic linkages, monosaccharide composition, functional groups, and the specificity of receptors [66,67]. This led to the activation of plant defense mechanisms in different ways.

Plant surface receptors (PRRs; pattern recognition receptors) are able to recognize carbohydrates (poly/oligosaccharides) as MAMPs or PAMPs, such as chitin, peptidoglycan (PGN), and glucans, and trigger an immune defense called PAMP-triggered immunity (PTI) [65,66]. Important biological activities are mediated by the interaction of saccharide elicitors and their associated proteins. The specific types of proteins that bind with specific carbohydrates are classified as lectins, which play an important role as PRRs [66]. Plant PRRs are receptor-like kinases (RLKs) or receptor-like proteins (RLPs) associated with a ligand-binding ectodomain (ECD) and a single-pass transmembrane domain. ECDs contain leucine-rich repeats, lysine motifs (LysMs), lectin motifs, or epidermal growth factor (EGF)-like domains. LysM-RLPs/RLKs are also known to recognize carbohydrate ligands. For example, chitin elicitor receptor kinase 1 (CERK1) has been reported as a co-receptor induced by several carbohydrate elicitors [66]. On the other hand, the wall-associated kinases, or WAKs, are receptors for pectin and OGAs, which are activated during pathogen responses [68]. However, WAKs have recently been reported to bind with alginates in the presence of calcium and ionic conditions for the formation of Ca^2+^ bridges between oligos and polymers [69]. This conformation, known as “egg boxes”, is necessary for the perception system in plants [70]. 

It has been reported that cuticular and stomata barriers have the potential to influence the level of resistance induced by plant elicitors [67,71]. After foliar application, elicitors have to cross these leaf barriers to be perceived by the cell wall and plasma membrane [67]. The stomatal pathway allows the diffusion of high-molecular-weight carbohydrates (over 43 nm in diameter), while the cuticular pathway has size-exclusion limits. It is rather compatible with the diffusion of small molecules (approximately 2 nm in diameter) [67,72]. This could further explain the variable efficacy between polysaccharides and oligosaccharides. Therefore, the physicochemical characteristics of elicitors in aqueous solutions, such as solubility and pH, may have a major influence on the success of foliar uptake and signal transduction. Several studies have shown the ability of some carbohydrates to promote stomatal closure. Li et al. revealed that the application of oligochitosans on the epidermal cells of *Brassica napus* L. induces the closure of stomata, depending on the production of H_2_O_2_ and nitric oxide (NO) [73]. Moreover, the application of laminarin exhibited a significant reduction in stomatal opening on grapevine leaves, whereas sulfated laminarin showed no inhibitory effect, regardless of the concentration [71]. Nevertheless, the perception of polysaccharides and oligosaccharides by plants is extremely complex, and more research is required.

A complex of oligochitosans and oligopectates has successfully been released into the market for crop protection under the brand FytoSave^®^. This product has shown protective effects against the downy mildew of grapes and cucumbers by reducing the severity of the disease [74]. Stemicol^®^ is another commercial phytovaccine that is composed of chito-oligosaccharide as the active substance. It is characterized by a M_w_ of less than 3 kDa and a DP of 5–18 monomers [75]. As a result, oligoalginates and low-molecular-weight alginates could be efficient defense-inducing agents that are readily recognized by plant receptors, leading to rapid signal transduction.

## 3. Materials and Methods

### 3.1. Biological Materials

Campbell 33 tomato seeds were obtained from Technisem (Longué-Jumelles, France). Under greenhouse conditions, sterile seeds were germinated in peat substrate for 20 days and then transplanted into pots containing soil and peat (*v*/*v*) for 45 days.

Two brown algaes, *S. muticum* and *C. myriophylloides*, were collected from the intertidal zone of the station Sidi Bouzid, El Jadida, Morocco, in July 2019. The samples were washed to eliminate salts and residues, oven dried, and then milled to a fine powder. 

### 3.2. Preparation and Characterization of Sodium Alginates and Their Derivatives

As described previously, alginates (ALCM and ALSM) were extracted according to the Mazumber et al. method [76] with minor modifications [10]. Briefly, 200 mg of algal powder was treated with formaldehyde (2%; 35 volumes), and then an aqueous solution of HCl (pH: 2–3; 30 volumes; 60 °C; 3 h; 500 rpm) was added to the residue. The conversion of alginic acid to sodium alginate was carried out using an alkaline treatment (Na_2_CO_3_; 2–3%; 25 volumes; 80 °C; 3 h; 500 rpm). ALCM and ALSM were precipitated with ethanol (96%), purified using the filtration system Vivaflow 50R200 (Sartorius Lab Instruments, Goettingen, Germany), and then lyophilized.

The alginate polymers were hydrolyzed using a previously described method [10]. This method is based on the oxidation of ALCM and ALSM using hydrogen peroxide. Briefly, aqueous solutions of sodium alginates (1% *w*/*v*) were treated with H_2_O_2_ in a 1:1 (*w*/*w*) ratio (70 °C; 6 h; 300 rpm). After adding ethanol (96%; 1/10 *v*/*v*), the samples were purified and freeze dried to obtain the OACM and OASM fractions.

The different saccharides were previously characterized using high-performance size-exclusion chromatography coupled with online multi-angle laser light scattering (HPSEC-MALLS). Gas chromatography–mass spectrometry (GC–MS) and ^1^H NMR were performed for the alginate polymers in order to identify the monosaccharide composition and the mannuronate/guluronate (M/G) ratio, respectively [10]. Biochemical analyses were performed as well to determine the total and neutral sugars, uronic acid, sulfate, protein, and polyphenol contents [10]. 

The distribution of DPs was carried out using high-performance anion-exchange chromatography with pulsed amperometric detection (HPAEC-PAD). OACM and OASM were prepared at 10 g/L in Milli-Q water to be injected into the Dionex ICS-3000 system using a CarboPac PA-200 column (4 mm × 250 mm) fixed to a CarboPac PA-200 guard column (Dionex 4 mm × 50 mm). Mannuronic acid was used as a standard.

FTIR analysis was carried out using a VERTEX 70 FTIR instrument. After 50 scans at room temperature, the spectra were determined in the range of 500–4000 cm^−1^.

### 3.3. Elicitor Treatment and Biochemical Assays

Saccharide fractions were dissolved in distilled water at a final concentration of 1 g/L, and the pH was adjusted to 6. The aqueous solutions were then applied using a foliar spray of 20 mL to 45-day-old tomato seedlings. The control seedlings were treated with distilled water. After 0, 12, 24, 48, and 72 h, the upper leaves (n and n − 1) and roots of four plants were collected and ground in liquid nitrogen for subsequent analysis. The “n” and “n − 1” refer to the nth and (n − 1)th leaves on the stem, respectively, when counting from the top down.

Phenylalanine ammonia-lyase (PAL), phenolic compounds, and lignin content were determined as described in previous work [57]. PAL activity was estimated by measuring the absorbance of *trans*-cinnamic acid at 280 nm after 1 h of enzymatic reaction [57]. The results were expressed in µmol of *trans*-cinnamic acid/mg protein/h/mg of dry weight (DW).

Proteins were estimated based on the Bradford assay using bovine serum albumin (BSA) as a standard [77].

Total polyphenols were first extracted and purified. Briefly, the phenolic extracts were treated with petroleum ether to discard pigments and lipids. After adding metaphosphoric acid (2%) and ethyl acetate, the extracts were evaporated and dissolved in acetone (80%). The purified polyphenols were then assayed using the Folin–Ciocalteu method [57]. The findings were represented as µg of gallic acid/g of dry weight (DW).

Lignin contents were extracted and quantified by monitoring the absorbance of the formed thioglycolic acid–lignin complex at 280 nm [57]. Briefly, the root and leaf tissues were heated at 100 °C in the presence of thioglycolic acid (1 M) and HCl (2 M). After 8 h of incubation, the recovered pellets were resuspended in NaOH (1 M) and placed at 20 °C for 18 h. Hydrochloric acid (12 N) was then added to the supernatants, and the formed thioglycolic acid–lignin complex was dissolved in NaOH (1 M) [57]. The results were given in A_280_/g of dry weight (DW).

### 3.4. Statistical Analysis 

The data were analyzed using the ANOVA test in SPSS version 25.0 software. The findings are represented as the mean value ± standard error (SE) of four replicates. At a *p*-value of less than 0.05, differences were considered statistically significant.

## 4. Conclusions

In this study, alginates were extracted from two Moroccan brown seaweeds, *S. muticum* and *C. myriophylloides*. Low-M_w_ alginates were obtained by oxidative hydrolysis. The M_w_ of the different fractions ranged from 202 to 3 kDa. FTIR analysis confirmed that the structures of the oxidized alginates remained unchanged, indicating that depolymerization using H_2_O_2_ with a ratio of 1:1 (*w*/*w*) seems to be an adequate method for alginate modification. The application of the alginates and their derivatives showed their capacity to induce PAL activity, phenolic compounds, and lignin content in the leaves and roots of tomato seedlings. The low-M_w_ alginates exhibited an effective induction of PAL activity compared to their native polymers, supplemented by a strong accumulation of secondary metabolites in both roots and leaves. In light of the foregoing, these findings underline the need to create new functional elicitors in agriculture, such as oligoalginates and low-M_w_ alginates, through oxidative degradation while maintaining many of the physicochemical properties of the native polymer. Furthermore, the use of these molecules represents a promising solution in crop protection for green and sustainable agriculture.

## Data Availability

Not applicable.
